# Correction: Convergent evolution of animal and microbial rhodopsins

**DOI:** 10.1039/d3ra90016a

**Published:** 2023-03-06

**Authors:** Keiichi Kojima, Yuki Sudo

**Affiliations:** a Faculty of Medicine, Dentistry and Pharmaceutical Sciences, Okayama University Japan keiichikojima@okayama-u.ac.jp sudo@okayama-u.ac.jp

## Abstract

Correction for ‘Convergent evolution of animal and microbial rhodopsins’ by Keiichi Kojima *et al.*, *RSC Adv.*, 2023, **13**, 5367–5381, https://doi.org/10.1039/D2RA07073A.

The authors regret an incorrect version of Fig. 2 and 5 were included in the original article. The correct version of Fig. 2 and 5 is presented below.

**Fig. 1:**
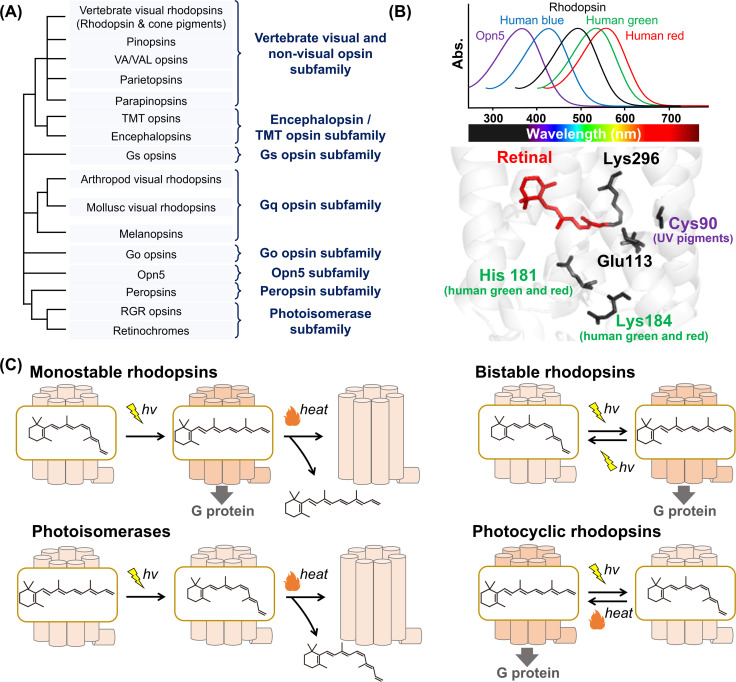
Phylogenetic relationship and the reaction of animal rhodopsins. (A) The phylogenetic tree of animal rhodopsins. Animal rhodopsins are roughly divided into 8 subfamilies.^7^ (B) Spectral sensitivities of animal rhodopsins, such as vertebrate rhodopsin, human blue-, green-, and red-sensitive cone pigments and Opn5m (upper panel). The crystal structure of bovine rhodopsin around the retinal (lower panel) (PDB: 1U19). The key residues for spectral tuning (His181 and Lys184 in human green- and red-sensitive cone pigments and Cys90 in vertebrate UV-sensitive cone pigments), retinal and Lys296, are highlighted in the structure. (C) The schematics of photoreaction of animal rhodopsins. In monostable rhodopsins, light irradiation induces isomerization from 11-*cis* to all-*trans*, thereby forming the active state. The active state bleaches followed by the release of retinal. In the bistable rhodopsins, light irradiation triggers interconversion between the inactive and active state containing 11-*cis* and all-*trans* retinal, respectively. Photoisomerases bind to the all-*trans* retinal exclusively in the dark state. Light irradiation of the dark state induces isomerization from all-*trans* to 11-*cis*. The product 11-*cis* retinal is delivered to regenerate other GPCR-type rhodopsins. The photocyclic rhodopsin Opn5L1 binds to all-*trans* retinal exclusively in the dark state. Light irradiation induces isomerization from all-*trans* to 11-*cis* to form the inactive state that is then thermally reconverted into the active state.

**Fig. 2:**
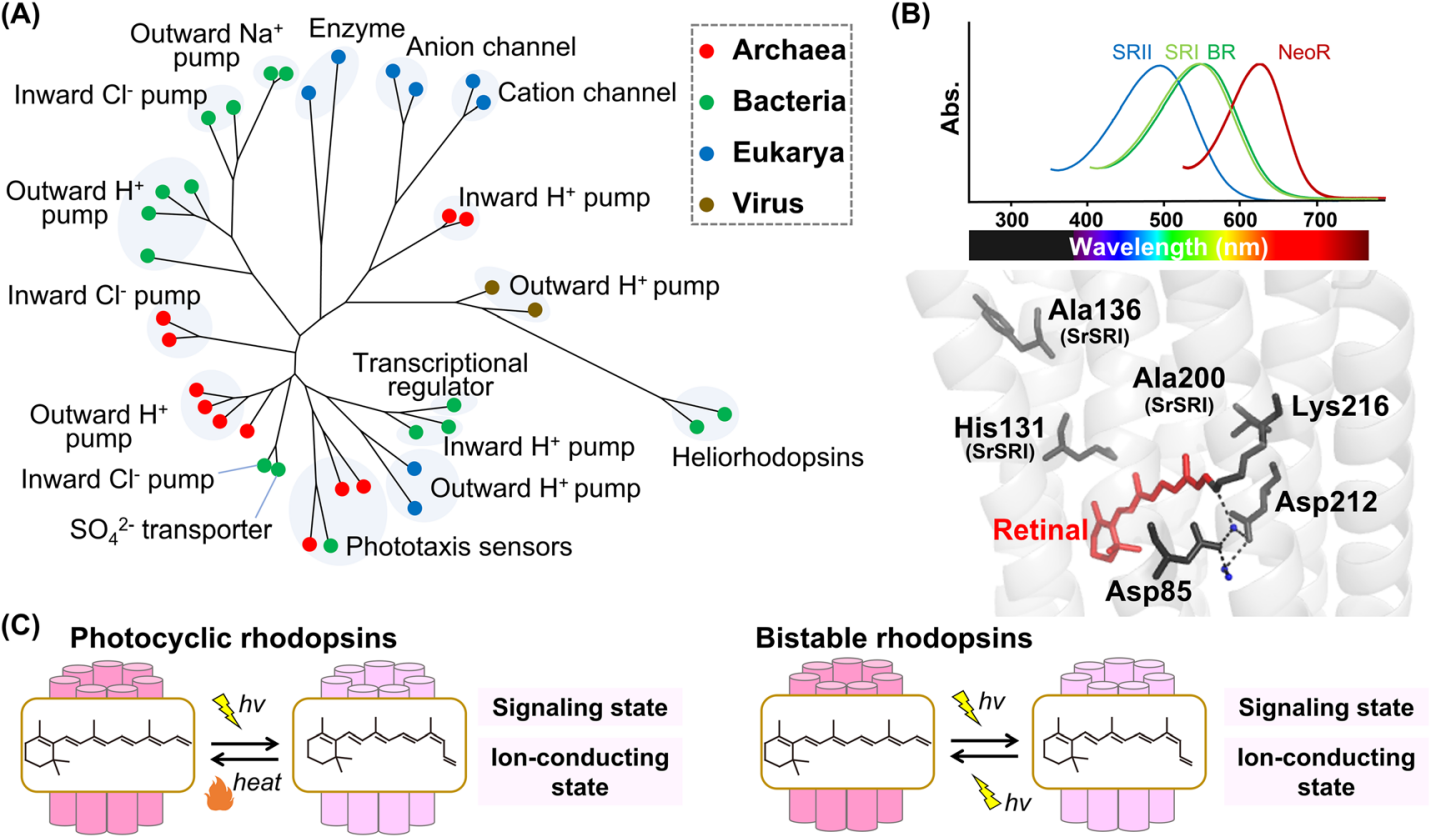
Phylogenetic relationship and reaction of microbial rhodopsins. (A) The phylogenetic tree of microbial rhodopsins. Rhodopsins from archaea, bacteria, eukarya, and viruses are indicated by filled circles colored red, green, blue, and brown, respectively. (B) Spectral sensitivities of microbial rhodopsins, such as BR, SRI, SRII and NeoR (upper panel). Crystal structure of BR around the retinal (lower panel) (PDB: 1C3W). The key residues for spectral tuning (His131, Ala136, and Ala200 in SRI from *S. ruber*, SrSRI), retinal, Lys216, and the pentagonal cluster (water molecules (blue dots), Asp85 and Asp212), are highlighted in the structure. The hydrogen-bonds of the pentagonal cluster are shown as black dashed lines. (C) The schematics of photoreaction of microbial rhodopsins. In the photocyclic rhodopsins, light irradiation induces isomerization from all-*trans* to 13-*cis* to form the signaling and ion-conducting states. The 13-*cis* retinal-binding form thermally returns to the initial all-*trans* retinal-binding form. Bistable rhodopsins show the thermally stable 13-*cis* retinal-binding form that is convertible to the initial all-*trans* retinal-binding form by illumination.

The Royal Society of Chemistry apologises for these errors and any consequent inconvenience to authors and readers.

## Supplementary Material

